# Structural Analysis of the German Self‐Report Questionnaire for Anxiety and Depression in People With Disorders of Intellectual Development (SAD‐IE)

**DOI:** 10.1002/cpp.70105

**Published:** 2025-06-23

**Authors:** Anika Gabriel, Almut W. Helmes, Charlotte T. Brecht, Julia I. Bräutigam, Markus A. Wirtz, Jürgen Bengel

**Affiliations:** ^1^ University of Freiburg Freiburg Germany; ^2^ University of Education Freiburg Germany

**Keywords:** anxiety disorders, confirmatory factor analysis, depressive disorders, disorders of intellectual development, SAD‐IE, self‐report questionnaire

## Abstract

**Background:**

Anxiety disorders and depression are among the most common mental disorders in people with disorders of intellectual development (DID). As many symptoms are not directly observable, diagnoses should not be based solely on third‐party assessment. The aim of the study was the development and structural analysis of a self‐report questionnaire (SAD‐IE) for anxiety and depression in people with DID.

**Methods:**

Based on the specific diagnostic criteria of DM‐ID‐2, a trial version of the SAD‐IE with 49 items was constructed in plain language. After contacting *N* = 233 institutions across all 16 German federal states, *N* = 286 adults with DID and their proxies were included. The factorial structure of the SAD‐IE was analyzed using confirmatory factor analysis for ordinal data (CFA, WLSMV estimation).

**Results:**

The CFA showed that a two‐factor model captured the latent structure of the SAD‐IE best (CFI = 0.956, TLI = 0.954, RMSEA = 0.040, SRMR = 0.069), with 39 of the original items having sufficient discriminatory power. The first factor, depression, was reliably represented by 22 items (*r* (it,c) = 0.520–0.770; *α* = 0.900, *ω* = 0.901) and the second factor, anxiety, by 17 items (*r* (it,c) = 0.504–0.796; *α* = 0.895, *ω* = 0.897).

**Conclusion:**

The German SAD‐IE is a reliable self‐report questionnaire measuring the major symptoms of anxiety and depression in people with borderline, mild, or moderate DID. It provides the foundation for an urgently needed self‐assessment in addition to existing third‐party assessments, ensuring a more comprehensive diagnosis. Further evaluation in practice is recommended.

**Summary:**

- The SAD‐IE is a self‐report questionnaire in German that can be completed independently or with little assistance by people with borderline or mild to moderate DID. Instructions and items of the SAD‐IE are written in plain language, and pictures for augmentative and alternative communication (AAC) are provided.
- The SAD‐IE takes into account the deviations in anxiety and depression in people with DID compared to the general population.
- The SAD‐IE includes relevant items to diagnose anxiety and depression, as well as two additional items to measure general psychological distress.
- The SAD‐IE is designed as a screening tool but could also be used to monitor subjective changes in symptoms during follow‐up assessments or in research.

## 1 Introduction

Mental health support for individuals with disorders of intellectual development (DID) represents one of the most pressing yet overlooked issues in modern healthcare. DID are characterized by significant limitations in both intellectual and adaptive functioning, affecting approximately 1%–2% of the general population (McKenzie et al. 2016). The ICD‐10 (Dilling et al. 2010) defines distinct DID severity levels ranging from mild (55 ≤ IQ ≤ 69) to profound (IQ ≤ 24) intellectual disabilities (ID), with each level presenting unique challenges in adaptive functioning and support needs. Additionally, individuals with borderline ID (70 ≤ IQ ≤ 84) face similar difficulties while not fully meeting formal DID diagnostic criteria. Adaptive functioning encompasses conceptual, social, and practical life skills. Studies indicate that mental health problems are up to four times more prevalent in individuals with DID (Janßen 2018; Einfeld et al. 2011), with anxiety and depression being among the most common disorders (Cooper et al. 2007). This increased vulnerability is often attributed to a combination of heightened risk factors and limited personal and social resources (Cooper et al. 2007). Despite this high prevalence, the mental health needs of people with DID often remain unmet, partly due to diagnostic challenges and limited adaptation of standard assessment procedures (Whittle et al. 2018; Costello and Bouras 2006).

With the ratification of the UN Convention on the Rights of Persons with Disabilities in 2009 and its further development in the German Federal Participation Act (Bundesteilhabegesetz) 2016, people with DID are guaranteed the right to equal access to mental health services. Nevertheless, third‐party assessments remain the standard procedure for diagnosing mental disorders in people with DID. Relying exclusively on this practice not only conflicts with the principles of self‐determined participation but also works particularly badly with highly internalizing disorders such as anxiety and depression, as many core symptoms elude external observation (Kremitzl et al. 2018; Mileviciute and Hartley 2015; Moss et al. 1996). Accordingly, these disorders are often underdiagnosed in (Lunsky et al. 2008).

The most common arguments against the application of self‐assessment tools for people with DID are comprehension problems, insufficient introspective abilities, and limited communication skills (Schmidt and Meir 2014). The more pronounced the DID and the lower the level of socio‐emotional development, the more these factors appear to represent a diagnostic obstacle. In addition, people with DID show deviations in symptom patterns compared to norm‐intelligent adults (Fletcher et al. 2016). Therefore, target group‐specific diagnostic manuals should be used to account for these differences in symptom manifestation. This is supported by Cooper et al. (2007), who demonstrated that conventional diagnostic systems (ICD‐10 and DSM‐IV‐TR) undercount mental health problems in this population. To obtain a valid self‐assessment, diagnostic tools must consider these specific characteristics of the target group as well. The Glasgow Anxiety Scale for people with an ID (GAS‐ID; Mindham and Espie 2003) and the Glasgow Depression Scale for people with a Learning Disability (GDS‐LD; Cuthill et al. 2003) are recognized as appropriate screening tools for anxiety and depression. However, these self‐report questionnaires lack certain features to promote comprehension, like pictures for AAC, and attempts to adapt them into German resulted in inadequate psychometric qualities (Müller et al. 2019; Müller et al. 2024). Therefore, we designed a single questionnaire for both anxiety and depression, as (1) these disorders are among the most prevalent in the target group (Cooper et al. 2007; Costello and Bouras 2006), (2) they occur as comorbidities of various other mental disorders (Brown et al. 2001; Michalak et al. forthcoming), and (3) their underlying constructs show high correlations (Masi et al. 2000; Nilges and Essau 2015). The significance of these disorders is underscored by findings from Cooper et al.'s (2007) comprehensive population‐based study (*N* = 1023): Clinical diagnoses of anxiety disorders (excluding specific phobias) showed a point prevalence rate of 3.8%. The prevalence was higher in individuals with mild ID (6.0%) compared to those with moderate to severe ID (2.4%). For affective disorders, clinical diagnoses showed a point prevalence rate of 6.6%.

The items of the SAD‐IE are based on the diagnostic criteria of the Diagnostic Manual—ID 2 (DM‐ID‐2; Fletcher et al. 2016). With increasing severity of DID and associated levels of socio‐emotional development, symptom presentations progressively differ from those observed in norm‐intelligent individuals (Duff et al. 1981; Došen 2005). While conventional diagnostic systems rely on concepts that do not account for developmental aspects in adults, the DM‐ID‐2 adapts its diagnostic criteria according to the severity of impairment. These adaptations include the addition or omission of symptoms, changes in symptom count and duration, and modifications in age requirements. On these grounds, we included (a) Separation Anxiety Disorder and Selective Mutism in the SAD‐IE anxiety scale and (b) additional symptoms (e.g., irritability) and behavioral equivalents to conventional symptoms (e.g., tearfulness) in the SAD‐IE depression scale. In general, we focused on basic emotions and easily accessible experiences. Accordingly, complex constructs such as derealization and depersonalization were not included in the SAD‐IE. An exception to this principle is the inclusion of the constructs of self‐esteem and self‐worth in the depression items, as empirical findings indicate, that people with DID are able to express problems in this area from a socio‐emotional developmental age of 7 years onward (Masi et al. 1999). A supplementary item was added to the SAD‐IE to capture general psychological distress. This item was not assigned to either of the two subscales.

The layout and language of the SAD‐IE were adapted to the German guidelines for plain language (Bundesministerium für Arbeit und Soziales 2022) and followed findings on methodological issues of questionnaire processing in people with DID (Finlay and Lyons 2001, 2002): The sentence structure was kept as short and simple as possible, with each line containing only one unit of meaning. Items were consistently phrased in question format, as it is more in line with the experience of people with DID to respond to questions rather than express agreement to statements. For example, the depression scale includes the item “Do things upset you easily?,” and the anxiety scale features the item “Are you scared when you ride the bus or train?” Double line spacing and a large, sans serif font were used. Each item was assigned a picture for ACC. The pictures were selected to reflect diversity and proximity to the everyday experiences of people with DID. In order to avoid fatigue and errors due to changing response categories, a three‐point Likert scale (“*yes*”—“*partly*”—“*no*”) was used throughout the questionnaire (Sonnenberg 2005). To facilitate interpretation, items were phrased so that agreement is associated with higher symptom expression. This is important because the SAD‐IE was developed as a screening tool that can be used not only by medical and psychotherapeutic staff but also by caregivers throughout the support system for people with DID.

Based on these methodological considerations, the aim of the present study was to develop and validate a screening questionnaire (SAD‐IE) for anxiety disorders and depression that people with DID can complete independently or with minimal assistance. While primarily designed for individuals with mild to moderate DID (35 ≤ IQ ≤ 69), we also included the often understudied population of people with borderline ID (70 ≤ IQ ≤ 84). Although a subset of people with borderline ID show high adaptive functioning, Peltopuro et al.'s (2014) systematic review revealed that the majority faces substantial challenges in areas such as cognitive skills, social information processing, and recognition of emotions, suggesting they too might benefit from the SAD‐IE's accessible design features. The present paper investigates three central aspects of the SAD‐IE's psychometric properties:

- the appropriateness of modeling the anxiety and depression items as two distinct but correlated latent constructs;
- the discriminatory power of individual items in relation to their underlying factors (anxiety or depression); and
- the reliability and internal consistency of the SAD‐IE's anxiety and depression scales.

## 2 Methods

The study was conducted in two project phases. In the first phase, a trial version of the SAD‐IE was developed and piloted. In the second phase, the SAD‐IE was tested and evaluated for its scale structure and psychometric properties as part of a cross‐sectional study.

### 2.1 Development of the SAD‐IE

The trial version of the SAD‐IE was developed in four steps: (1) item pool generation, (2) item reduction, (3) cognitive interviews, and (4) piloting. The item pool was generated based on validated clinical questionnaires for adults and children, supplemented by items developed in expert panels. The items (*n* = 116 for anxiety, *n* = 156 for depression) were clustered according to the core criteria for anxiety disorders and depression of the DM‐ID‐2 (Fletcher et al. 2016). The item reduction was an iterative process in which the item pool was discussed and gradually reduced in five panels with *N* = 13 experts from the fields of clinical research, psychotherapy, psychiatry, special education, and special needs assistance for people with DID. The reduction process stopped as soon as the expert panel agreed on a maximum of two item wordings per criterion. The items and accompanying texts were translated into plain language by employees with and without DID from a certified bureau for plain language. They also offered validated pictures for AAC, which the research team subsequently adapted for use in this questionnaire. Additionally, where no existing pictures were available, custom pictures were commissioned. Semi‐structured cognitive interviews based on an adaptation of Tourangeau's model of survey response (Tourangeau 1984; Jen‐Yi et al. 2015) were conducted with *N* = 5 participants with DID using the think‐aloud technique (Pohontsch and Meyer 2015). The participants' feedback was systematically recorded (Ridolfo and Schoua‐Glusberg 2011) and incorporated into the trial version of the SAD‐IE. The trial version of the SAD‐IE contained 49 items. In the pilot phase, the feasibility of the trial version was tested under survey conditions with *n* = 6 participants with DID, and recommendations for assistance were derived.

### 2.2 Procedures

Data collection for the second phase of the study took place between January 2022 and September 2023. After contacting *N* = 233 facilities across all 16 German federal states by telephone and email, *N* = 63 facilities agreed to participate in the study. The facilities included psychotherapeutic outpatient practices and clinics, medical centers for adults with DID, assisted living services, and sheltered workshops for people with DID. The test leaders on site (usually staff members such as psychologists or sheltered workshop managers) were instructed to approach potential participants, their proxies, and, if applicable, their legal guardians, to provide them with information about the study. All study materials for people with DID were written in plain language. Participants were eligible for inclusion if they were adults with a diagnosed or suspected borderline, mild, or moderate DID. Additional criteria were adequate German language skills and the presumable ability to comprehend and respond to questions about their own behavior and experiences in plain language, either independently or with little support. Recruitment aimed at a diverse sample, including individuals with and without comorbid mental and personality disorders. Only those currently experiencing psychotic episodes or engaged in substance misuse (except tobacco) were excluded. The study also required the participation of a proxy who had known the person with DID for a minimum of 6 months and maintained regular contact. This proxy agreed to provide third‐party assessments. After all parties involved had given their written consent, the surveys began. Test leaders on site received a reimbursement of 85 Euros per completed dataset, which, in most cases, they passed on to the participants with DID.

### 2.3 Sample

Of *N* = 328 subjects with DID who had initially agreed to participate in the study, *n* = 42 data sets had to be excluded, which corresponds to a drop‐out rate of 12.8% and a final sample of *N* = 286. The reasons for exclusion were: (a) termination of study by the test leader on site (*n* = 11), by the participants (*n* = 6) or by the proxies (*n* = 3); (b) acute psychosis (*n* = 8); (c) active substance use disorder (*n* = 2); (d) no borderline, mild or moderate DID with onset before adulthood (*n* = 7); (e) language barrier on the part of the proxy (*n* = 1); (f) no regular contact between participant and their proxy within the last 4 weeks (*n* = 2); and (g) violation of the 14‐day period between the conduction of the SAD‐IE and the Mini PAS‐ADD Interview (*n* = 2). We stopped the recruitment due to time restraints and the knowledge that a sample size of *N* = 286 has sufficient power to meet the statistical requirements for all intended analyses. The average age of the *N* = 286 included participants with DID was 40.91 years (SD = 13.4, Md = 38) and ranged from 18 to 77 years. The gender distribution showed that *n* = 130 participants were female, *n* = 125 male, and *n* = 1 identified as diverse. Regarding the severity of DID, *n* = 59 participants had borderline DID, *n* = 160 participants had mild DID, and *n* = 67 participants had moderate DID. Across all severity levels of DID, participants were predominantly living in assisted living environments or residential care facilities rather than independently without professional caregiver support. The majority of participants with borderline DID lived in supported independent living arrangements, while those with mild or moderate DID most frequently lived in residential care facilities. As expected, educational attainment and employment in the primary labor market tended to be higher among participants with lower DID severity levels. Table 1 presents detailed demographic characteristics of the sample, stratified by DID severity level.

**TABLE 1:** Demographic characteristics of the sample.

	Severity of DID			
	Borderline *n* = 59	Mild *n* = 160	Moderate *n* = 67	Total *N* = 286
*Age, M, (SD)*	40.4 (13.9)	40.1 (12.6)	43.4 (14.7)	40.9 (13.4)
*Gender, n, (%)*				
Male	24 (40.7%)	74 (46.3%)	27 (40.3%)	125 (43.7%)
Female	35 (59.3%)	85 (53.1%)	40 (59.7%)	160 (55.9%)
Diverse	—	1 (0.6%)	—	1 (0.3%)
*Living situation, n, (%)*				
Living alone	6 (10.2%)	7 (4.4%)	—	13 (4.5%)
With parents/relatives	3 (5.1%)	8 (5.0%)	8 (11.9%)	19 (6.6%)
With partner	1 (1.7%)	5 (3.1%)	—	6 (2.1%)
Supported independent living	22 (37.3%)	43 (26.9%)	17 (25.4%)	82 (28.7%)
Supported group living	10 (16.9%)	20 (12.5%)	3 (4.5%)	33 (11.5%)
Residential care facility	13 (22.0%)	69 (43.1%)	37 (55.2%)	119 (41.6%)
Other	4 (6.8%)	8 (5.0%)	2 (3.0%)	14 (4.9%)
*Highest level of education, n, (%)*				
Still in school	—	2 (1.3%)	—	2 (0.7%)
No degree	6 (10.2%)	32 (20.0%)	17 (25.4%)	55 (19.2%)
Special education	28 (47.5%)	108 (67.5%)	43 (64.2%)	179 (62.6%)
Elementary school	20 (33.9%)	15 (9.4%)	3 (4.5%)	38 (13.3%)
Secondary school diploma	4 (6.8%)	—	—	4 (1.4%)
Other	—	3 (1.9%)	4 (6.0%)	7 (2.4%)
Missing	1 (1.7%)	—	—	1 (0.3%)
*Current work situation, n, (%)*				
Employment in primary labor market	11 (18.7%)	10 (6.3%)	1 (1.5%)	22 (7.7%)
Employment in supported work environments	36 (61.1%)	115 (71.9%)	50 (74.7%)	201 (70.3%)
In vocational training	2 (3.4%)	1 (0.6%)	—	3 (1.0%)
Retired	4 (6.8%)	12 (7.5%)	8 (11.9%)	24 (8.4%)
Unemployed	3 (5.1%)	4 (2.5%)	2 (3.0%)	9 (3.1%)
Other	3 (5.1%)	18 (11.3%)	6 (9.0%)	27 (9.4%)

### 2.4 Measures

#### 2.4.1 Baseline Survey

In the baseline survey, proxies provided sociodemographic information on the participants with DID. The collected variables included age, gender, living situation, the highest level of education, the highest level of vocational training, current work situation, diagnosed mental disorders, and current treatment. Additionally, the duration and nature of the relationship between the participants with DID and their proxies were documented.

#### 2.4.2 Level of ID Assessment Sheet

To verify whether the inclusion criteria of borderline or mild to moderate DID (35 ≤ IQ ≤ 84) were met, the proxies also completed the Level of ID Assessment Sheet (Koch et al. 2017). It assesses the functional aspects of spoken language, reading, writing and arithmetic, autonomy, activities of daily living, occupational skills, and interpersonal skills. The questionnaire defines the overall severity of DID on five levels: borderline, mild, moderate, severe, or profound. In addition, it inquires about diagnoses of DID in medical records, its etiology, and the age of onset.

#### 2.4.3 Trial Version of the SAD‐IE

The trial version of the SAD‐IE contained a total of *N* = 49 items. Of these, *n* = 27 items assessed the core symptoms of depression, and *n* = 21 items assessed universal anxiety symptoms such as avoidance and safety behaviors, as well as specific core symptoms of the following disorders: Separation Anxiety Disorder, selective mutism, specific phobia (subtypes: blood–injury, animals, darkness, and heights), panic disorder, agoraphobia, social anxiety disorder, and generalized anxiety disorder (GAD). A supplementary item, which was not assigned to either of the two scales, inquired about general psychological distress. As this item was not included in the calculations, the analysis of the scales was based on *n* = 48 items. The participants with DID completed the SAD‐IE as independently as possible, but in the presence of an assistant. The assistant received instructions on how to support adequately the participant in the event of comprehension difficulties or queries. Immediately after processing the questionnaire, the assistant wrote down the completion time, any notable incidents, and the type and level of help provided in an administration protocol. This documentation served two purposes: to provide guidance for future questionnaire administrators and to verify that the average completion time remained within 30 min, considering the limited attention span of individuals with DID.

#### 2.4.4 Mini PAS‐ADD Interview

Finally, within 14 days after the SAD‐IE was completed, the Mini PAS‐ADD Interview (Moss 2002; German version: Weber and Fritsch 2003) was conducted with the proxies. It is a semi‐structured interview for assessment by caregivers that screens for a broad spectrum of mental disorders in adults with DID, including depression, anxiety, hypo (mania), obsessive‐compulsive disorder, and psychosis. In the present study, the German‐language adaptation (Weber and Fritsch 2003) was used, and supplemented with questions on alcohol and substance use disorders. As recommended by the authors, an assessment period of 4 weeks was chosen. The interviews were conducted by psychologists who were at least in training as psychological psychotherapists. They received intensive training and continuous supervision. The interviews were conducted by telephone and lasted approximately 50 min. The outcomes of the Mini PAS‐ADD Interview were used to check the exclusion criteria for acute psychosis and substance use disorders and to test the construct validity of the final version of the SAD‐IE. The latter is not the subject of this article and will be reported elsewhere.

### 2.5 Data Analyses

After the missing data analyses, a confirmatory factor analysis (CFA) was conducted using the Weighted Least Squares Mean Variance (WLSMV) estimator in Mplus Version 8.8 (Muthén and Muthén 2007) to examine the factorial structure of the questionnaire. The WLSMV estimator is based on the nonparametric assumption of ordinal data. It was used to address potential measurement artifacts associated with the three‐point Likert scale response format, such as expected floor and ceiling effects and deviations from normal distribution (Brauer et al. 2023).

Based on theoretical considerations, a two‐factor model with the factors “depression” and “anxiety” was assumed to fit the item structure best. In addition, a one‐factor model was calculated to test whether the proposed two‐factor solution exhibits a superior fit. Several fit indices were used for the evaluation of the goodness of fit (Schermelleh‐Engel et al. 2003): chi‐square Statistic, Comparative Fit Index (CFI; values ≥ 0.95 acceptable; ≥ 0.97 good), Tucker–Lewis Index (TLI; values ≥ 0.95 acceptable; ≥ 0.97 good), Standardized Root‐Mean‐Square Residual (SRMR; values ≤ 0.08 acceptable; ≤ 0.05 good), and Root‐Mean‐Square Error of Approximation (RMSEA; values ≤ 0.07 good; Hooper et al. 2008).

To optimize the model fit of the two‐factor model, items with loadings < 0.50 were removed. The final model was then further tested at a local level. Significance and standardized loadings *λ*_*i*_ (criterion > 0.63) and the indicator reliability (criterion > 0.40) were determined for each item. The psychometric suitability of the individual survey scales was tested using the Average Variance Extracted (AVE; criterion > 0.50) and factor reliability (criterion > 0.60; Kline 2023). The internal consistency of the two subscales was tested using Cronbach's alpha and McDonald's omega, with values of ≥ 0.70 considered acceptable and ≥ 0.80 considered good (George and Mallery 2019).

## 3 Results

### 3.1 Missing Data Analyses

Missing data analyses revealed that among the *N* = 286 participants, a total of *n* = 36 missing values were identified, with a maximum of *n* = 4 missing values per item. There was no evidence of systematic patterns in the missing data, including any associations with age, gender, or other participant characteristics. The missing values were imputed using the Expectation–Maximization algorithm in SPSS version 29 (Schafer and Graham 2002).

### 3.2 Confirmatory Factorial Analysis of the SAD‐IE

The examination of the CFA fit indices showed a better fit of the two‐factor model compared to the one‐factor model, with values of 0.954 for the CFI, 0.952 for the TLI, and 0.074 for the SRMR (see Table 2). The nested model comparison revealed a chi‐square difference of Δ*χ* ^2^ (df = 1) = 175.505 in favor of the two‐factor model. These results suggest that the information in the SAD‐IE items is better explained by two separate factors, namely “depression” and “anxiety” than by a single‐factor solution.

**TABLE 2:** Goodness of fit indices of the CFA models.

Model	Number of items	$\chi^{2}$	*df*	*p*	CFI	TLI	SRMR	RMSEA [95% CI]
One‐factor model	48	1611.141	1080	< 0.001	0.932	0.929	0.082	0.041 [0.037; 0.046]
Two‐factor model	48	1435.637	1079	< 0.001	0.954	0.952	0.074	0.034 [0.029; 0.039]
Model comparison (Δ)	48	175.504	1	< 0.001	0.022	0.023		
Final two‐factor model	39	1019.554	701	< 0.001	0.956	0.954	0.069	0.040 [0.034;0.045]

In the second step, a total of *n* = 9 items was excluded from the two‐factor model due to loadings *λ*_*i*_ < 0.50. These were five items initially assigned to the factor “depression”: Item 3 (indifference, *λ*_*i*_ = 0.399), Item 5 (aggressiveness toward objects, *λ*_*i*_ = 0.406), Item 6 (aggressiveness toward oneself/others, *λ*_*i*_ = 0.460), Item 12 (increase in appetite, *λ*_*i*_ = 0.336), and Item 24 (thoughts of death, *λ*_*i*_ = 0.466); and four items assigned to the factor “anxiety”: Item 35 (specific phobia blood and injection type, *λ*_*i*_ = 0.377), Item 36 (specific phobia animal type, *λ*_*i*_ = 0.374), Item 37 (specific phobia environment type darkness, *λ*_*i*_ = 0.442), and Item 38 (specific phobia environment type heights, *λ*_*i*_ = 0.430). The two‐factor model with reduced items showed the best model fit (see Table 2), with the greatest incremental improvement in the SRMR (0.069). Subsequently, this model was chosen as the final model for the SAD‐IE.

The two factors “depression” and “anxiety” proved to be highly correlated (*r* = 0.71), which corresponds to the typical findings in the literature (Kühner et al. 2007; Nilges and Essau 2015). Nevertheless, according to the Fornell–Larcker criterion, the two factors were reliably separable, since the square of this correlation (*r* ^2^ = 0.50) was smaller than both the AVE = 0.64 of the depression factor and the AVE = 0.69 of the anxiety factor (Kline 2023). No further item reduction was implemented to increase the separability of the modeled factors because the individual scale properties were very good.

### 3.3 Scale Characteristics of the SAD‐IE Depression and Anxiety Scales

Table 3 shows analyses for all individual items. In the final model, the factor loadings were generally high (*λ*_min_ = 0.504, *λ*_max_ = 0.796). The final model was further supported by the local goodness of fit, as indicated by the item‐total correlations with satisfactory scale properties (discriminant validity: *r*_min_ = 0.408; *r*_max_ = 0.678), and by the good to excellent values for the internal consistency of the subscales (depression: *α* = 0.900, *ω* = 0.901; anxiety: *α* = 0.895, *ω* = 0.897), indicating a high reliability of the subscales.

**TABLE 3:** Item and scale statistics of the SAD‐IE questionnaire.

Item	Frequency			M (SD)	Loading	Item‐total‐correlation	Internal consistency	
	no	partly	yes				*α*	*ω*
	0	1	2					
*Depression*							0.900	0.901
1 Sadness	91	128	67	0.92 (0.74)	0.708	0.576		
2 Tearfulness	136	104	46	0.69 (0.73)	0.601	0.474		
4 Irritability	60	103	123	1.22 (0.77)	0.521	0.432		
7 Loss of interest	177	73	36	0.51 (0.71)	0.510	0.450		
8 Diminished pleasure	148	88	50	0.66 (0.76)	0.617	0.546		
9 Emotional numbness	169	77	40	0.55 (0.73)	0.573	0.447		
10 Loss of skills	136	72	78	0.80 (0.84)	0.567	0.485		
11 Social withdrawal	161	72	52	0.62 (0.78)	0.678	0.557		
13 Loss of appetite	190	60	36	0.46 (0.71)	0.539	0.430		
14 Disrupted sleep	127	88	71	0.80 (0.81)	0.607	0.510		
15 Tiredness	71	111	104	1.12 (0.78)	0.547	0.485		
16 Psychomotor agitation	108	106	72	0.87 (0.79)	0.679	0.523		
17 Psychomotor retardation	159	77	50	0.62 (0.77)	0.603	0.488		
18 Exhaustion	127	59	100	0.91 (0.89)	0.682	0.534		
19 Lack of energy	123	98	65	0.80 (0.79)	0.652	0.563		
20 Reduced drive	164	73	49	0.60 (0.77)	0.566	0.490		
21 Low self‐esteem	151	89	46	0.63 (0.75)	0.758	0.602		
22 Low self‐worth	153	67	66	0.70 (0.82)	0.770	0.610		
23 Concentration problems	128	106	52	0.73 (0.75)	0.688	0.587		
25 Suicidality	248	32	6	0.15 (0.42)	0.626	0.416		
26 Rumination	120	82	84	0.87 (0.84)	0.659	0.505		
27 Hopelessness	147	70	69	0.73 (0.83)	0.726	0.534		
*Anxiety*							0.895	0.897
28 Intense anxiety	142	78	66	0.73 (0.81)	0.792	0.667		
29 Inappropriate anxiety	181	56	48	0.53 (0.77)	0.776	0.585		
30 Avoidance	169	73	44	0.56 (0.75)	0.609	0.487		
31 Safety behavior	140	59	87	0.81 (0.87)	0.531	0.469		
32 Fear of separation	157	66	63	0.67 (0.82)	0.677	0.579		
33 Fear of loss	129	68	89	0.86 (0.86)	0.504	0.408		
34 Selective mutism	115	75	96	0.93 (0.86)	0.648	0.498		
39 Rapid panic onset	196	50	40	0.45 (0.73)	0.679	0.551		
40 Physical panic reaction	134	62	90	0.85 (0.87)	0.736	0.636		
41 Fear panic recurrence	166	55	65	0.65 (0.83)	0.778	0.678		
42 Agoraphobia public transportation	219	45	22	0.31 (0.61)	0.556	0.419		
43 Agoraphobia enclosed spaces	205	34	47	0.45 (0.76)	0.640	0.476		
44 Agoraphobia crowds	173	61	52	0.58 (0.78)	0.727	0.607		
45 Agoraphobia open spaces	194	43	49	0.49 (0.77)	0.528	0.442		
46 Social anxiety	109	86	91	0.94 (0.84)	0.778	0.590		
47 GAS free‐floating	98	87	101	1.01 (0.84)	0.796	0.591		
48 GAS uncontrollability	135	83	68	0.77 (0.81)	0.787	0.593		

*Note:* All loadings were significant *p* < 0.001.

## 4 Discussion

In previous studies, our research group has already made attempts to provide clinically applicable self‐report questionnaires for anxiety and depression in people with DID (Müller et al. 2019; Müller et al. 2024). These efforts included adapting the English Glasgow Scales (GAS‐ID, Mindham and Espie 2003; GDS‐LD, Cuthill et al. 2003), but they did not present acceptable psychometric properties. For the development of the current questionnaire, we took a fundamentally new approach, creating the instrument from scratch rather than adapting existing measures. While this approach required significant time and resources, it resulted in an evidence‐based self‐assessment screening tool that addresses the special needs of people with DID from a multi‐professional perspective.

### 4.1 Modeling and Factor Structure

The structural analysis of the SAD‐IE confirmed the assumed two‐factor structure of the SAD‐IE: The CFA showed that the two‐factor model with the latent factors “depression” and “anxiety” had a better fit compared to a one‐factor model with a single superordinate latent factor. The model fit was optimized by excluding items with factor loadings < 0.50. Therefore, the CFA resulted in a reduction of the SAD‐IE from its trial version of 48 items to a final version containing a total of 41 items.

### 4.2 Discriminatory Power and Selection of Individual Items

Item‐level analyses showed that the remaining items demonstrated a strong alignment with their intended scales. In this final version, the SAD‐IE anxiety scale comprises 17 items, which represent anxiety disorder‐overarching symptoms as well as disorder‐specific core criteria. Notably, all four excluded anxiety items with factor loadings < 0.5 were items that operationalized different subtypes of specific phobia (subtypes: blood–injury, animals, darkness, and heights). As a result, the final version of the SAD‐IE does not provide indications of specific phobia. Beyond this statistically driven decision, theoretical considerations also suggest the need for a dedicated questionnaire for diagnosing specific phobias in this population: Individuals with DID typically struggle with transfer and generalization tasks (Lifshitz et al. 2011). The feedback in our administration protocols illustrated this challenge, revealing instances where users with animal phobias failed to appropriately affirm Item 36 when they feared other animals than those explicitly mentioned in the examples. Addressing this would require an exhaustive list of specific phobic stimuli, a task beyond the scope of a broad screening tool like the SAD‐IE, especially considering the limited concentration capacity of its users (Laßmann et al. 2019).

The depression scale was reduced to a total of 22 items in the final version of the SAD‐IE due to loadings < 0.5 for five items. For most items (indifference, increase in appetite, and thoughts of death), the exclusion does not pose a content‐related problem, as the operationalized symptom criteria are sufficiently covered by other items. However, the joint exclusion of Items 5 (aggression toward objects) and 6 (aggression toward self/others) presents a clinical concern. For individuals with DID, irritability and aggressive behavior are key features of depressive disorders and are thus included among the core criteria of DM‐ID‐2 (Fletcher et al. 2016). Yet, research indicates that while self‐ and other‐directed aggression in people with DID is more prevalent in comorbid depression, it is not exclusive to it. Aggressive behavior appears to be an expression of general distress (Crocker et al. 2014; Moss et al. 2000). This may explain why Items 5 and 6 in the trial version of the SAD‐IE loaded more strongly on the depression factor but also substantially on the anxiety factor. Consequently, we chose to remove both aggression items from the depression scale. However, we retained the original Item 6 on self‐ and other‐directed aggression as a supplementary item in the SAD‐IE. Together with the supplementary item on psychological distress, it can provide information on the general psychological burden of the users with DID beyond the domains of anxiety and depression. Completely omitting the assessment of aggressive behavior in the SAD‐IE would be unsound from a clinical perspective, as self‐ and other‐directed aggression usually indicates a need for protective measures for the affected person with DID or their social environment and requires further diagnostic clarification.

### 4.3 Reliability and Internal Consistency of the Scales

The final two‐factor model was further supported by additional analyses of the local goodness of fit, which revealed satisfactory scale properties and an excellent internal consistency of the subscales with Cronbach's alpha at 0.900 and McDonald's omega at 0.901 for depression and 0.895 and 0.897 for anxiety.

### 4.4 Development Strategy and Key Features

We posit that our multi‐step strategy in developing the questionnaire was crucial to the success of the SAD‐IE, which we attribute to the following key factors:

1. The operationalization of anxiety and depression was based on criteria from the target group‐specific DM‐ID‐2 diagnostic manual (Fletcher et al. 2016), allowing for a straightforward transfer of SAD‐IE test results to diagnosis.
2. We integrated systematically evaluated feedback from expert panels, comprising professionals from diverse disciplines, including psychotherapy, psychiatry, special education, and clinical research.
3. We engaged in an iterative process to balance the questionnaire's diagnostic requirements with language comprehensibility for people with DID. This latter step was facilitated by bureaus specializing in plain language, ensuring the questionnaire remained accessible to its target population. This collaboration resulted in the certification of the SAD‐IE in plain language. Individuals with DID themselves reviewed the content and user‐friendliness of the questionnaire as part of the bureaus' assessments and through cognitive interviews. This participatory approach allowed people with DID to directly influence the design of the SAD‐IE as lived experience experts.
4. The SAD‐IE incorporates validated AAC pictures for both its instructions and individual items. These pictures exhibit high iconicity (i.e., visual resemblance to their referents) and are tailored to the daily life experiences of the target group. The research team selected and modified the pictures to ensure alignment with the clinical content.
5. In determining the basic design criteria, we incorporated findings from research on perception, information processing, and communication in individuals with DID (Finlay and Lyons 2001; Laßmann et al. 2019). The literature suggests a 30‐min maximum concentration span for people with DID (Laßmann et al. 2019). Contrary to initial concerns, the 49‐item trial version of the SAD‐IE did not prove overly challenging, even for participants with moderate DID. The average completion time was 23.69 min (SD = 13.48). Administration protocols indicated that interruptions during the assessment process were rarely necessary. Extended completion times frequently resulted from participants' desire to elaborate on their responses to the assisting person. Interestingly, many individuals with DID reported that completing the SAD‐IE was not a burden, but an opportunity to express their own feelings and thoughts in a one‐on‐one setting.
6. A distinctive feature of the SAD‐IE is its use of a three‐point Likert scale, instead of the five‐point Likert scale, which is commonly used in other psychological self‐report questionnaires. Response formats with three instead of five categories were found to be more suitable for people with DID (Fang et al. 2011). At the same time, the reduced number of response options increased the risk of measurement artifacts, such as floor and ceiling effects. To address this, we employed the WLSMV estimator for ordinal data. Results from structural analyses indicate that the response scale has sufficient discriminatory power.
7. The trial version of the questionnaire was conducted on a large sample of *N* = 286 individuals with borderline, mild, or moderate DID. This exceptionally large sample size for our target population ensures sufficient statistical power to detect existing effects.

### 4.5 Limitations

While the present study demonstrates promising results, it is important to acknowledge two main limitations that should be considered when interpreting these findings. First, the selection of cooperating institutions and participants did not follow a randomized or stratified process. Instead, participation was based on voluntary engagement, which may involve self‐selection processes. Furthermore, specific exclusion criteria were applied (e.g., current psychosis or substance use). Therefore, this sample cannot be considered representative of the general population of people with DID in Germany, and no statements can be made about how the measured characteristics are distributed in this population. Nevertheless, we expect our results to be widely applicable because our study participants and survey conditions closely match real‐world care practices: We recruited participants in clinical settings (e.g., outpatient psychotherapy practices) as well as non‐clinical settings (e.g., assisted living services and sheltered workshops for people with DID) across all 16 German federal states. Staff members at these facilities helped administer the questionnaire, which is important because the SAD‐IE was designed for these specific settings and users. To enhance generalizability, the inclusion criteria allowed both, individuals without mental health issues and those with comorbidities across the entire spectrum of mental disorders and personality disorders to participate in the study. We only excluded people with active substance use disorders or those experiencing a psychotic episode during data collection, as these conditions can severely affect perception. The descriptive analysis showed that individuals of female and male gender were equally represented in the sample, as were individuals with borderline, mild, or moderate DID. The age range spanned from 18 to 77 years.

Second, we decided to use the Level of ID Assessment Sheet (Koch et al. 2017) for the classification of DID severity levels of the participants. The use of standardized test batteries to determine individual IQ scores could have led to a more precise classification. However, we deliberately decided against this approach for several reasons. Mainly because IQ tests are time intensive and must be conducted by trained diagnosticians. Most of our cooperation partners would not have been able to fulfill these requirements. Moreover, a previous study by our research group showed that psychometric IQ testing represents a far‐reaching intrusion into the participants' privacy, which raises ethical concerns (Müller et al. 2019). In line with this approach, many comparable studies have opted against psychometric IQ testing and used clinical judgment or information from patient records instead to determine the severity of DID (Hamers et al. 2019; Hermans et al. 2013).

### 4.6 Conclusions and Future Directions

In conclusion, the SAD‐IE is the first self‐report questionnaire for anxiety and depression in people with DID in plain language and with pictures for ACC. Content‐wise, the questionnaire closely follows the target group‐specific diagnostic criteria according to DM‐ID‐2. At the same time, its design emphasizes high user‐friendliness. As a result, individuals with borderline, mild, or moderate DID can complete the SAD‐IE independently or with minimal support. Assistance requires no special training. The SAD‐IE demonstrates good reliability in clinical and non‐clinical samples. To help a widespread use, we publish the questionnaire free of charge. Given these factors, we firmly believe that the SAD‐IE has the potential to fill a current void in the psychotherapeutic care system. Further, the development process of the SAD‐IE could serve as a blueprint for self‐report questionnaires addressing other disorders, such as obsessive‐compulsive disorders or post‐traumatic stress disorders as well as clinically relevant aspects of psychological well‐being, including self‐esteem and life satisfaction.

## Ethics Statement

This study protocol was reviewed and approved by Ethik‐Kommission Universitätsklinikum Freiburg, approval number (21‐1208). The study complies with the Helsinki Declaration.

## Consent

Written informed consent was obtained from all participants. Participants with disorders of intellectual development received all documents in plain language. Where applicable, consent was also obtained from their legal guardians.

## Conflicts of Interest

The authors declare no conflicts of interest.

## Data Availability

Due to the particular vulnerability of the target group, data are not shared publicly. The corresponding author will provide information about the dataset used in this study upon request.
